# Supplementary material for: A Review of Registered Randomized Controlled Trials for the Prevention of Obesity in Infancy
Source: Int J Environ Res Public Health. 2021 Mar 2;18(5):2444. doi: 10.3390/ijerph18052444 (PMC7967587; doi:10.3390/ijerph18052444)
Supplement: Supplementary file 1 [file ijerph-18-02444-s001.pdf]

Supplementary files

Table S1. Characteristics of early intervention studies for the prevention of obesity in infancy (N=29).

	Registration No	Trial Name/Acronym	Ref no<br><br>Author, Year (if published)	Country	Study design	Number randomized	Intervention commencement	Duration of follow up	Primary outcome(s)	Secondary outcome (s)	Delivery mode	Intervention components	Controls
	ACTRN12607000168459	Healthy Beginnings	Wen 2012<br><br>Wen 2015	Australia	RCT	N=667 mother-child dyads	Antenatally	Birth until 5 years	Child BMI z score at child age 2 and 5 years	Infant feeding practices, TV viewing time, active play time, mothers dietary behaviours	Nurse home visits	The nurse visited eight times at home, once at 30-36 weeks' gestation and seven times after the birth (at 1, 3, 5, 9, 12, 18 and 24 months). Four key areas included infant feeding practices, child nutrition and active play, family physical activity and nutrition, and social support.	Usual care: 1x Community Health nurse home visit plus home safety promotion materials at 6 and 12months.
2.	ISRCTN81847050	InFANT	Campbell 2013	Australia	Cluster RCT	N= 542 parent-child dyads	Mean 3.8 months	Child age 4months until	Child diet (3x 24 hour diet recalls), child physical	BMI z scores	Sessions with dietitian in pre-existing mothers groups, supportive	Six 2 hour sessions of education delivered by dietitian targeting	Usual care with Maternal and Child Health nurse plus

								20 months	activity (accelerometry) and child TV viewing (parent report)		materials (DVD, written materials and newsletter)	nutrition, physical activity and sedentary behaviours occurred within pre-existing mothers groups, commencing at 3 months at 3monthly intervals (3, 6, 9, 12, 15 and 18 months). Didactic sessions, group discussion and peer support. Reinforced by purpose designed DVD and written materials plus newsletter between sessions	6 newsletters regarding unrelated aspects of child health or development
3.	ACTRN12608000056392	NOURISH	Daniels 2013<br>Daniels 2015	Australia	RCT	N=698 mother-child dyads	Child age 4 months	Child age 4 months until 5 years	BMI z score at 24months and 5 years	Maternal feeding practices	Educational group sessions in supported by detailed written information	Two educational group session modules (6 fortnightly sessions each) commencing at infant age 4-7months and again at 13-16months supported by detailed written information regarding	Usual care: standard community child health services

											repeat food exposure to variety of foods, responsive feeding and cues, health child food intake, reduce TV viewing, promotion of authoritative parenting style, managing food fussiness	
4.	NCT00756626	Feeding Young Children Study: Bottle Weaning Interventio n (FYCS)	Bonuck 2013	USA	RCT	N=300 parent-child dyads	12months of age	Child age 12 months until 24 months	Bottle use frequency	Anthropometric measurements, age and sex specific weight-for-length. Dietary intake and nutrient density	Educational counselling, education materials and a incentive sippy cup	Conducted at WIC; Educational counselling about bottle feeding via a flip-chart was given to parents (with messages about healthy weight, dental caries and iron deficiency anaemia), an education pamphlet to share with family members and a sippy cup was provided. Baseline anthropometric measurements (height and weight), sociodemograp hic survey and dietary intake	Usual care: Routine visits to Women, Infants and Children's (WIC) sites

												assessment were all completed at baseline (12months), 15months, 18months, 21months and 24months	
5.	NCT00892983	POI.nz	Fangupo 2015<br><br>Taylor 2016<br><br>Taylor 2018	New Zealand	2x2 factorial RCT	N=802 parent-child dyads	Antenatally	Antenatally to 2 years of age (follow up study has data at 3.5 and 5 years of age)	Weight velocity (0-6, 6-12, 12-24months) and BMI z scores at 24months	Sleep and physical activity (parent report, accelerometry), duration of breastfeeding, timing of solid introduction, diet quality, measures of family function and well being	Home visits and group sessions	3 intervention groups. FAB (Feeding, Activity and Breastfeeding): 8 additional visits for education and support around breastfeeding, food and activity (breastfeeding focus at antenatal and 1 weeks of age, food related at 4,7,13 and 18months and physical activity at 3,9 and 18months age) provided by lactation consultants and trained research nurses. Reinforced with written and visual information. SLEEP: 2 additional	Usual care: Routine Well Child checks (6 core visits in the first 18months)

												reviews providing guidance and resources home sleep (group antenatal session and home visit at 3 weeks). Combination: both (i.e. 9 intervention visits/sessions	
6.	NTR1831	The BeeBOFT Study	van Grieken 2017	Netherlands	Cluster RCT	N=2102 parent-child dyads	1month of age	Child age 1 month to 36 months	Health-related behaviour (breakfast daily, activity and outside play, sweetened beverage consumption, television viewing and computer time), BMI and prevalence of overweight and obesity at 36 months of age	None listed	Web-based eHealth module and discussion of personalised advice during regular well child visit	E-health4Uth Healthy Toddler- parents completed an online module which provided personalised education regarding their childs nutritional habits and physical activity at 18months and 24 months. This was followed by face-to-face counselling by the YHC professional to parents during routine well child visits. Parents completed questionnaires regarding	Usual care: Routine well baby visits at 18 and 24 months with general information on child health-related behaviours was provided to parents

											family characteristics and health-related behaviours when child 1month, 6months, 14months and 36months of age	
**7.**	**NCT01040897**	GREENLIGHT	Sanders	USA	RCT	N= 865 parent-infant dyads	Child age 2months	Child age 5months to 2 years	BMI at 2 years	Child dietary intake, physical activity and injury prevention behaviours	Tool kit with written information, routine reviews with trained Paediatric resident in primary care clinics	Low-literacy toolkit for parents, a health-communication curriculum for child-health providers, including modules on goal-setting techniques, educational toolkit, a clinician-centred curriculum for providing low-literacy guidance on obesity prevention. Behaviour-change components administered by Paediatric residents at each well-child visit from 2	Usual care: plus, injury prevention program (attention placebo)

										months to 24 months.	
8.	NCT03370445	Addressing Health Literacy and Numeracy to Prevent Childhood Obesity	Cruzatt	USA	RCT	N= 450 parent-child dyads (estimate)	Child age 2 months	Child age 2months to 5 years	Proportion of children at 24months who are not overnight (BMI)	Weight status of children at 5 years of age (BMI z score)	'Tool kit' with written information, routine reviews with trained Paediatric resident in primary care clinics	Interaction with Paediatric resident physicians who are trained in improved health communication skills. Also provided with low literacy handouts and study-related 'gifts' to assess nutrition and physical activity behaviours and reinforce evidence based recommendations about early childhood nutrition and physical activity	Usual care: plus injury prevention program (attention placebo)
9.	NCT01167270	INSIGHT	Savage 2016<br>Adams 2018<br>Savage 2018	USA	RCT	N= 279 mother-child dyads	Child age 1-2 weeks	Child age 1-2 weeks to 3 years	BMI at 3 years	Patterns of infant weight gain, infant sleep duration, maternal responsiveness, maternal feeding style, infant dietary content and physical activity.	Mailed educational visits, trained nurse home visits, research centre visits and phone calls to deliver messages	At 2 weeks post -partum, intervention materials were mailed to the participant's home. Research nurses conducted home visits at 3, 16, 28, and 40 weeks, and a research centre	Usual care: Routine care plus trained research nurse home visits, research centre visits, phone

			Paul 2018									visit occurred at 1 year. parents are taught how to identify and respond to infant hunger and satiety cues and are also educated on growth charts	calls that delivered home safety intervention (strategies to prevent Shaken Baby Syndrome and child abuse, treatment of fever and other first aid remedies, fire, bath, and car seat safety are discussed.)
1(	NCT01198847	Early STOPP	Sobko	Sweden	RCT	N= 200 families	Child age 1 year	Child age 1 year to 6 years	BMI at 6 years	Physical activity, sedentary behaviour, motor function/development, sleeping habits, food intake, eating patterns and quality of life.	Trained coach (dietitian, physiotherapist or a nurse), phone calls, mailed information, home visits, clinic visits, booklets	One educational and one individually targeted coaching. The educational material (booklets) is developed for correspondent age (1, 2, 3, 4, 5 and 6 years) and is given to the parents. The coaching is delivered by a trained coach four times the	Usual care: Routine Child Health Care Clinic plus general health newsletter at baseline

											first year and twice a year after which takes place in the family's home, or at the clinic.	
11	ACTRN12611000386932	INFANT Extend	Campbell	Australia	Cluster RCT	N= 540 mother-child dyads	Child age 3months	Child age 3 months to 36 months	BMI z score and waist circumference at 36 months	Dietary quality, physical activity, screen time	Online educational content, emailed newsletters, nutrition expert	Melbourne InFANT Program content will be delivered via six emailed newsletters (3 monthly from child age 18 months to 3 years Educational content will be made available online. First-time parent group will be mediated by a nutrition expert, for up to one hour per week.	Usual care: Routine care in Maternal and Child Health Centres plus general health newsletters ever three months for three years
12	NCT01541761	Starting Early Obesity Prevention Program	Gross	USA	RCT	N= 533 women-child dyads	Antenatally	Antenatally to 3 years (published data exists at 3 months)	Reduction in prevalence and degree of obesity at 3years (BMI percentiles), diet composition, infant lifestyle	(2x 3month published data: Infant feeding practices and maternal infant feeding knowledge (study 1) and infant activity time (Study 2)	Individual nutrition/breast feeding counselling, educational curriculum in group sessions at well baby visits supported by handouts and DVDs	Individual nutrition/breast feeding counselling, 15 nutrition and parenting support groups (NPSG) coordinated with well child visits, supported with	Usual care: Standard prenatal visits then standard Paediatric primary care (at 5days, 1month, 2months

									behaviours (better sleep habits, reduced screen time, increase physical activity), improvement in parent feeing knowledge attitudes, styles and practices.			plain language handouts and nutrition education DVDs. (Visits: prenatal consultation on breastfeeding, lactation support on postnatal ward, then 15 group sessions at 1,2,4,6,9,12, 15,18,21,24,27,30 and 33 months)	and 4months) then
1[illegible]	NCT01649115	HLPP	Reddy	USA	RCT	N=150 parent-child dyads	Antenatally	Birth to 5 years	Change in child's gender-specific WFL z-score at 4 to 6 months of age	BMI at child age 2 and 5 years, Mothers Knowledge,	Two face to face meetings with registered dietitian and once on phone, nutritional education handout	The first meeting, they will be receiving the Healthy Lifestyles Passport, including the interactive nutrition education. The second meeting is a post-test assessment and Newest Vital Sign Assessment Tool.	Usual care plus meeting with registered dietitian twice and once on home with standard care information and handouts
1[illegible]	ACTRN12612001133820	Baby-led introduction to solids (BLISS)	Taylor 2017	New Zealand	RCT	N= 206 women	Antenatally	Antenatally until child age	Body Mass Index at 12months (and follow-up	Energy self regulation	Home visits, face to face meetings and phone calls with lactation	8 additional contacts from pregnancy to 9 months of age. 5 meetings with	Usual care: routine midwifery

				24months	at 24 months)	Eating behaviours Energy intake	consultant and trained researcher.	lactation consultant antenatally, at 1 week, 3-4 weeks, 3-4months and 5 months (3x face to face and 2x via telephone,10-60mins duration) to support prolonged milk feeding and delay of complementary feeding until 6months. Followed by face to face meetings (30-60min) with trained researcher at 5.5, 7and 9 months with individualised advice to support to assist mothers with above plus further education on appropriate foods and feeding cues. Questionnaires on baby led approach adherence at 6, 7, 8, 9, 12, and 24 months. BMI	and well child care

												calculated at 12 and 24 months. Secondary outcomes assessed via questionnaires at 12 and 24 months.	
15	NCT01905072	Preventing Childhood Obesity through Early Guidance	Reifsnider 2013	USA	RCT	N=140 pregnant women	Antenatally	Child age 1 week to 3 years	BMI at 3 years	Age of solid food introduction and dietary intake, Breast/Bottle feeding practices, Sleep, Screen time	Child health care worker home visits, phone calls, Women Infant and Children's (WIC) clinic	Monthly phone calls regarding breastfeeding status. Home visits at 36 weeks of pregnancy; at 3 days after birth; at 2 weeks of age; and at 2, 4, 6, 9, 12, 18, and 24 months. Printed materials with key concepts; growth monitoring, feeding, parenting, activity, and sleep.	Usual care: Routine WIC clinic care plus home visits to monitor measurements and monthly breastfeeding survey calls
16	ISRCTN16991919	PRIMROSE	Doring, 2016	Sweden	Cluster RCT	N=1369 infants (1355 families)	Child age 9-10months	Child age 9-10 months to 4 years	Four-year-old children's BMI and Waist Circumference	Mother and child eating habits (FFQ), Mother and child physical activity (accelerometer/validated questionnaire), Mothers' BMI & waist circumference.	Intervention operates within Child Healthcare Centres, 5 sessions with CHC trained nurses on child health behaviours	Nurses assist parents to change their own health behaviours and to promote healthy dietary and physical activity behaviours in their children. Parents are	Usual care: Regular age-related health check ups

												offered individual session when child is 9-10 months old, then group and phone sessions at 1.5,2,3, and 4 years old.	
1[illegible]	PMC4442409	Early Obesity Prevention	Schroeder, 2015	USA	Cluster RCT	N= 232 infants	Paediatric visit at 1 month of age	All paediatric visits at 1, 2, 4, 6, 9, 12, 15, 18, and 24 months of age, and at annual visits up to age 5 years	BMI, BMI Z score, triceps skinfolds, weight	Parental dietary practices, breastfeeding duration, SNAP participation	Face to face, during all paediatric visits up till 24 months. Supportive phone call once a month and reminder post cards with short educational messages	Families receive 'growing leaps and bounds' program which includes : nutrition, physical activity, feeding practices, eating patterns, enhancing parents self-efficiency to care for infants, helping parents make healthy food choices for their infants and themselves<br>The 12 sets of educational brochures are discussed at 1, 2, 4, 6, 9, 12, 15, 18, and 24 months paediatric visits	Usual care: standard paediatric visits
1[illegible]	ACTRN12616001470482	CHAT	Wen 2017	Australia	RCT (3 arm)	N=1056 mother-child dyads	Antenatally	Third trimester to child	BMI z-score at 12 and 24 months, breastfeedi	Child TV viewing time at 12 and 24 months; dietary	Mailed educational material,	Intervention arm 1 (SMS): Text messages providing information	Usual care: Routine childhood nursing

								age 1 year	ng rate at 12 months, and timing of introduction of the solids at 6 months	quality at 24 months	telephone or SMS support	about healthy infant and child feeding and lifestyle twice a week for 4 weeks. Intervention arm 2 (telephone support) 6 staged intervention packages by mail followed by a phone call from the research nurse within 1–2 weeks nurse and discuss the issues or concerns raised by the mother.	services from Community Health Services plus home safety promotion material and newsletter on Kids Safety 4 times over the study period
1[illegible]	NCT03077425	CHALO	Karasz, 2018	USA	RCT	N=360 mothers of children 4-6 months old	Home visits at 6 months of age	Children aged 6 months to 18 months	Quantity of sippy cups/ bottles, number of sippy cups/bottles a day (My smile buddy)	Weight for length, BMI for age Z score, weight velocity Z score, added sweeteners/solids, fruit and vegetables/day, sweetened beverages, use of bottles/sippy cup at bed time, sweet and salty snacks, physical activity, screen time, tooth brushing, dental visits, caries	Home visits by community health workers, follow up telephone support, patient navigation to keep timely dental visits, pamphlets	n=6 home visits by health works: four of the home visits include only mother-child dyads which focus on building rapport, intimacy, identifying risks or family concerns, education, discussing goals, enhance	Enhanced usual care, pamphlet containing ECC and obesity prevention messages. and dental referrals,

												mother skills to identify infant hunger/satiety cues, and oral hygiene practices.<br><br>Two of the home visits at 8 and 14 months included either the father or mother in law	
2[illegible]	NCT03131284	Prevention of Obesity in Toddlers (PROBIT) Trial	Morandi 2019	Italy	RCT	N=529 parent-newborn dyads	First 2 weeks of life	Newborn to 2years of age	BMI at 24 months of age	BMI at 12months, lifestyle and feeding practices in the first 2 years of life	Educational program by Paediatricians during routine visits	Paediatricians were trained to provide parents with standardized lifestyle counselling supported by educational written material about the first two years of life during routine visits at 1, 3, 6, 12 and 24 months.	Usual care: Routine visits with regular Paediatrician at 1, 3, 6, 12 and 24 months
2[illegible]	NL6727<br><br>(NTR6938)	"Samen Happie!" An mHealth intervention to prevent obesity through parenting	Karssen L 2017	The Netherlands	RCT	N= 300 parent-child dyads (estimate)	7-11months	7-11months until 4 years	Child BMI at 6months, 12months to 4 years.	Parental parenting weight-related behaviours (eating, drinking, sleeping, physical activity/screen time) Weight-related behaviours of the child (healthy eating, drinking,	Mobile application	Mobile application that teaches parents about health parenting practices and styles, but also allows parents to practice through various challenges. The information is grouped into	Waitlist control condition (receive the app after the 12month intervention period)

										sleeping, and exercise)<br>General parenting style<br>Parental wellbeing (e.g., including depression, self-reported health, happiness)<br>Parental cognitions (e.g., including parenting related self-efficacy and motivation)		four important determinants: eating, drinking, sleeping and exercise	
2[illegible]	NCT03334266	Preventing Early Childhood Obesity, Part 2: Family Spirit Nurture, Prenatal - 18 Months	Ingalls 2019	USA	RCT	N=338 expectant mothers (estimate)	Antenatally	Antenatal (36 weeks) until child age 24months	BMI z scores<br>Breast and complementary feeding rates<br>Implementation of infant and toddler responsive feeding behaviours<br>Consumption of fruits and vegetables<br>Calorie intake from sugar sweetened beverages (SSB),	Maternal stress<br>Maternal depression<br>Maternal alcohol and drug use<br>Infant and maternal metabolic health	Home visits by trained Family Health Liaison	36 comprehensive lessons delivered biweekly from 28 weeks until birth, weekly from birth until 3months, bi weekly from 3 to 6months, monthly from 6months to 18months.	Standard Optimised Standard of Care (OSC) transportation assistance to prenatal appointments and well-baby clinic appointments PLUS 8 home visits with educational lessons on injury prevention topics (attention placebo)

									snacks, and desserts, Physical activity levels. Screen time and other sedentary activities				
23	NCT03348176	Baby's First Bites: Promoting Vegetable Intake in Infants and Toddlers	Van der Veek 2019	Netherlands	RCT factorial	N= 240 mother-child dyads	Child age 4 months	Child age 4 months until 36 months	Vegetable consumption, vegetable liking and self-regulation of energy intake	Child eating behaviours, child anthropometrics (BMI) and maternal feeding behaviour	Phone calls, home visits, video feedback	3x intervention arms. Repeated vegetables exposure (RVE)- exposure to either green beans or cauliflower as target vegetable during first 19days of weaning. 5x phone calls to motivate parents to exposure children at 4-6, 8, 13 and 16months. Video feedback Intervention to promote Positive Parenting Feeding of infants (VIPP-FI)- exposure to fruits and sweet vegetables during first	Usual care: plus attention control condition- 5x phone calls with mother about development of child, no advice on complementary feeding

												19days of weaning, 5x home visits using video feedback to promote sensitive feeding at 4-6, 8, 13 and 16months. COMBI-RVE+VIPPFI	
24	NCT03399617	SPOON: Guatemala	Gonzalez 2018	Guatemala	RCT	N= 1500 care giver-child dyads (estimate)	0-6months	0-6months until 24months	Infant and young child feeding practices<br>Child Height<br>Weight gain rate<br>Haemoglobin<br>Prevalence of obesity in children (BMI) Prevalence of stunting Prevalence of anaemia	Adherence to Nutritional Supplement Regime,<br>Exclusive Breastfeeding	Home visits, group sessions and community mobilisation activities, dietary supplement	2x intervention groups: SPOON behavioural change strategy+SQ-LNS: Participants will receive Small Quantity Lipid-based Supplements (SQ-LNS) from 6-24 months and a behavioural change to promote adequate infant and young child feeding practices and the use of SQ-LNS will be delivered to mothers or caregivers. SPOON behavioural change strategy+MNPs:	Usual care: Participants receive standard health care services provided by the Ministry of Health, including micronutrient powders (MNPs)

												Participants will receive micronutrient powders (MNPs) from 6-24 months and a behavioural change to promote adequate infant and young child feeding practices and the use of MNPs will be delivered to mothers or caregivers. Behaviour change strategy uses ethnographic and marketing methods to promote adequate infant and young child feeding practices and the use of SQ-LNS	
25	NCT03438721	Strong Futures	Beck 2018	USA	RCT	N=240 parent-child dyads (estimate)	Child age 2weeks	Child age 2 weeks until 24months	Child dietary intake<br>Child screen time<br>Parent health-related	Child anthropometrics (BMI z score at 6, 12, 15 and 24months)<br>Parent financial stress<br>Child sleep	Education program during well child visits	Parents will receive education on infant feeding, sleep, and screen time practices just after well-child visits in the first year of life (2 weeks, 2, 4, 6, 9	Usual care: plus financial coaching; education on financial topics i.e. budgeting, savings, managing

									quality of life	Parental feeding styles		and 12months). The education will be provided by a lay health educator. Parents will also receive text messages to reinforce the intervention content	debt (provided by lay health educators trained in financial coaching), reinforced by text messages
2[illegible]	NCT03444415	PROGESPI	Perez-Lopez 2018	Spain	Cluster RCT	N=414 parent-child dyads (estimate)	Antenatally	Antenatally until 24months age	BMI at 24months	Weight growth rate, Food intake habits in parents, physical activity in parents, Smoking habit in parents, Anthropometry of parents (BMI), duration of breastfeeding, Child dietary habits, Physical activity patterns in children, Sleep habits, sociodemographic variables	Motivational Interviewing in groups	6x 90minute group workshops (2x during pregnancy, 4x within two years after birth) by trained researchers (GP, nurses, Paediatricians and midwives) to encourage healthy lifestyles to parents, encourage breastfeeding and increase knowledge and self-efficacy to promote health habits re diet, physical activity and sleep habits	Usual care: General information about height, weight and BMI percentile by health professionals during well child visits

27	ChiCTR1800017773	SCHeLTI (Sino-Canadian Healthy Life Trajectory Initiative)	Wu 2018	China	Cluster RCT	N=4000 mother-child dyads (estimate)	6 weeks	6 weeks until 5 years	BMI at 5 years	Fat mass index (child) at 5 years, skinfold thickness at 5 years, birthweight for gestational age prior to hospital discharge after delivery, weight-for-length z-score at <2 years of age	Face to face education sessions, group educational activities, text messaging, motivational web-based tools and apps, community based activities	Multifaceted intervention aiming to positively change behaviour with patient centred face to face sessions, group educational activities and social supports, text messaging to encourage personal goals and barriers to behaviour changes, motivational web-based tools and apps for self monitoring and community based activities	Usual care: plus access to web-based tools and Apps that provide information on child health and safety.
28	NCT03752762	SPOON-Mexico	Martinez 2018	Mexico	RCT	N=1200 care giver-child dyads (estimate)	0-6months	0-6months until 24months	Infant and young child feeding practices Height Weight gain rate<br>Haemoglobin Prevalence of obesity Prevalence of stunting<br>Prevalence of anaemia	Adherence to Nutritional Supplement Regimen, Exclusive Breastfeeding	Home visits, group sessions, dietary supplement	SQ-LNS supplement from 6-24 months and an innovative behavioural change strategy designed using ethnographic and marketing methods to promote adequate infant and young child feeding practices and the use of SQ-LNS via home	Usual care: Standard health care services as specified by the Health Secretary

												visits and group sessions. Data recorded at child age 6, 9, 12, 15,18, 21 & 24 months	
29	NCT04042467	Greenlight plus study(55)	Rothman 2019	USA	RCT	N=900 parent-infant dyads	First newborn clinic visit	All child Dr visits from 0-24 months	Child weight for length trajectory	Weight for length Z score, BMI Z score trajectory, child overweight or obese	During all child clinic visits from 0-24 months	Families will receive the Greenlight intervention plus a health information technology (HIT) intervention aimed at supporting family goal-setting and behavior change. They will receive instructions on how to access the Greenlight technology platform (iOTA text messaging and website) Families will consistently receive text messages and goals set for children in the first two years of life	During child's clinic visits from 0 -24 months, parents will the receive the basic Greenlight material (low literacy, age specific education booklet) to promote healthy lifestyle and obesity prevention

FAB: Feeding Activity Breastfeeding, NPSG:Nutrition Parenting Support Groups, LNSP- SQ: Lipid Nutrient Supplement Paste Small Quantity, MNP: Micronutrient Powders, POI: Prevention of Obesity in Infancy, SNAP: Supplemental Nutrition Assistance Program , WIC: Women, Infants, and Children Special Supplemental Nutrition Program, YHC: Youth Health Care

Table S2. Intervention delivery materials and procedures of early intervention studies for the prevention of obesity in infancy (N=29).

**Intervention Delivery**	**Materials**								**Procedures**		
**Trial name**	**Educational handout**	**Educational handout (image-based)**	**Educational video**	**Low literacy educational tool kit**	**Educational website/app**	**Educational material mailed out**	**Feeding supplement**	**Nutrition and parenting support groups**	**Phone call consultation**	**Home Visits**	**Educational text messages**
**TOTAL N (%)**	**15(52)**	**4(14)**	**2(7)**	**3(10)**	**5(17)**	**5(17)**	**2(7)**	**7(24)**	**6(21)**	**12(41)**	**4(14)**
Healthy Beginnings	x										
InFANT	x										
NOURISH	x							x			
FYCS	x										
POI.nz	x	x						x		x	
BeeBOFT						x				x	
GREENLIGHT	x			x							
Addressing Health Literacy and Numeracy to Prevent	x			x							

Childhood Obesity									
INSIGHT					x			x	
Early STOPP	x				x		x	x	
InFANT EXTEND				x	x				
Starting Early Obesity Prevention Program		x	x			x			
HLPP	x						x		
BLISS	x	x						x	
Preventing obesity through early guidance	x						x	x	
PRIMROSE								x	
Early Obesity Prevention:	x			x					
CHAT		x			x		x		x
CHALO	x						x	x	
PROBIT	x								
Samen Happie				x					
Family Spirit Nurture								x	

Baby's first bites			x						x	x	
SPOON-Guatemala							x	x		x	
Strong Futures											x
PROGESPI								x			
SCHeLTI					x			x			x
SPOON-Mexico							x	x		x	
Greenlight plus	x			x	x						x

Table S3. Intervention delivery agent.

**Intervention Delivery Agent**<br><br>**Trial name**	**Nurse home visits**	**Nurse clinic visits**	**Registered Dietitian**	**Lactation consultants**	**Trained Research assistants**	**Community Health Worker**	**Community Health Worker**<br><br>**Home visits**	**Nutrition expert**	**Paediatric residents/Paediatrician**	**Psychologist**	**General Practitioners**	**Midwives**	**Physiotherapist**	**Trained Sleep Specialists**	**Multidisciplinary**
**Total N(%)**	**5 (17)**	**5 (17)**	**5 (17)**	**3 (10)**	**4 (13)**	**1 (3)**	**4 (13)**	**2 (7)**	**4 (13)**	**1(3)**	**1(3)**	**1(3)**	**1(3)**	**1(3)**	**2(7)**
Healthy Beginnings	x														
InFANT		x	x												
NOURISH			x							x					
FYCS			x												
POI.nz	x			x										x	
BeeBOFT						x									
GREENLIGHT									x						
Addressing Health Literacy and Numeracy to Prevent Childhood Obesity									x						
INSIGHT	x														
Early STOPP	x	x	x										x		

InFANT EXTEND							x		
Starting Early Obesity Prevention Program				x			x		
HLPP			x						
BLISS				x	x				x
Preventing obesity through early guidance						x			
PRIMROSE		x							
Early Obesity Prevention		x						x	
CHAT	x								
CHALO						x			
PROBIT								x	
Samen Happie									
Family Spirit Nurture					x				
Baby's first bites					x				
SPOON-Guatemala						x			

Strong Futures		x					
PROGESPI	x			x	x	x	
SCHeLTI							x
SPOON-Mexico			x				
Greenlight plus				x			

Table S4. Intervention components/key messages of early intervention studies for the prevention of obesity in infancy (N=29).

Trial name/acronym	Breast feeding/Bottle feedng advice	Intro of solids	Limit junk foods (eg sweets)	Repeat food exposure	Healthy dietary behaviours in children	Food serving size	Parenting / hunger satiety cues	Parent modelling	Fussy eating	Soothe / Sleep	Sleep promotion	Play/ activity	Tummy time	TV/ screen time	Oral hygiene practices	Growth chart education	HIT technology access education	Health-communication curriculum
**Total N (%)**	**16 (55)**	**10 (34)**	**6 (21)**	**3 (10)**	**24 (83)**	**5 (17)**	**13 (45)**	**13 (45)**	**5 (17)**	**3 (10)**	**10 (34)**	**20 (69)**	**3 (10)**	**9 (31)**	**1 (3)**	**1 (3)**	**1 (3)**	**2 (7)**
Healthy Beginnings	x	x	x		x		x	x	x	x		x		x				
InFANT					x		x	x	x			x		x				
NOURISH				x	x		x	x										
FYCS	x																	
POI.nz	x	x			x						x	x						
BeeBOFT			x		x							x		x				
GREENLIGHT	x				x			x			x	x		x				x
Addressing Health Literacy and Numeracy to Prevent Childhood Obesity	x				x			x			x	x		x				x
INSIGHT				x	x		x		x	x	x	x				x		
Early STOPP					x						x	x						
InFANT EXTEND					x		x	x	x			x		x				
Starting Early Obesity Prevention Program					x	x	x	x	x	x	x	x						
HLPP					x	x												
BLISS	x	x					x											
Preventing obesity	x		x				x					x						

through early guidance																	
PRIMROSE					x			x				x					
Early Obesity Prevention		x	x		x	x	x	x				x					
CHAT	x	x			x	x		x				x	x	x			
CHALO	x	x					x					x	x		x		
PROBIT	x	x	x		x	x	x					x					
Samen Happie	x				x						x	x					
Family Spirit Nurture	x		x		x		x	x				x	x	x			
Baby's first bites		x		x			x										
SPOON-Guatemala	x	x			x												
Strong Futures					x						x			x			
PROGESPI	x				x			x			x	x					
SCHeLTI	x				x						x	x					
SPOON-Mexico	x	x			x												
Greenlight plus					x			x									x

Table S5. Theoretical Models.

Theoretical models used<br><br>Trial name	Social cognitive theory	Social-ecological theories	Social learning theory	Health beliefs model	Behavioural model	Attachment theory	Transtheoretical model of change	Ecological developmental theory	Theory of planned behaviour	McGuire communication model	Common Risk/Health Factor Approach (CR/HFA)	Not listed
**TOTAL N (%)**	**10(34)**	**1(3)**	**5(17)**	**3(10)**	**1(3)**	**2(7)**	**1(3)**	**1(3)**	**1(3)**	**1(3)**	**1(3)**	**16(55)**
Healthy Beginnings			x	x								
InFANT	x											
NOURISH	x					x						
FYCS												x
POI.nz												x
BeeBOFT	x	x	x						x	x		
GREENLIGHT	x											
Addressing Health Literacy and Numeracy to Prevent Childhood Obesity	x											
INSIGHT												x
Early STOPP	x				x		x					
InFANT EXTEND												x
Starting Early Obesity Prevention Program	x		x	x								

HLPP							x
BLISS							x
Preventing obesity through early guidance							x
PRIMROSE	x						
Early Obesity Prevention:							x
CHAT		x	x				
CHALO						x	
PROBIT							x
Samen Happie							x
Family Spirit Nurture	x				x		
Baby's first bites	x	x		x			
SPOON-Guatemala							x
Strong Futures							x
PROGESPI							x
SCHeLTI							x
SPOON-Mexico							x
Greenlight plus							x

Table S6. Funding Sources.

Study	Funding Sources
Healthy Beginnings	Australian National Health and Medical Research Council
INFANT	Australian National Health and Medical Research Council
NOURISH	Australian National Health and Medical Research Council<br>HJ Heinz<br>Meat and Livestock Australia<br>Department of Health South Australia<br>Food standards Australia New Zealand<br>Queensland University of technology<br>Roberta Holmes Transition to contemporary Parenthood Program (La Trobe University)
Feeding Young Children Study	US department of Agriculture<br>National Institute of Food and Agriculture
POI.nz	Health Research Council New Zealand<br>Southern District Health Board
BeeBOFT	ZonMW- Netherlands Organisation of Health Research and Development<br>NOW- Netherlands organisation for scientific research
GREENLIGHT	Eunice Kennedy National Institute of Child Health and Human Development<br>Centers for Disease Control and Prevention<br>Office of Behavioural and Social Science Research<br>National Institutes of Health<br>Robert Wood Johnson Foundation Physician Faculty Scholars Program<br>Health Resources and Services Administration<br>KiDS of the New York University Langone Foundation
Addressing Health Literacy and Numeracy to Prevent Childhood Obesity	NYU Langone Health<br>National Institutes of Health

INSIGHT	National Institute of Diabetes and Digestive and Kidney Diseases (NIDDK)<br>National institute of Health<br>National Center for Advancing Translational Sciences<br>The Children's Miracle Network (Penn State Children's Hospital)<br>US Department of Agriculture<br>Penn State Clinical and Translational Institute
Early STOPP	FAS<br>Vinnova<br>Medical Research Council<br>The Karolinska Institute
INFANT extend	Australian National Health and Medical Research Council<br>National Heart Foundation<br>World Cancer Research Fund International
Starting Early Obesity Prevention Program	National Institute of Food and agriculture<br>National institute of Health/Child Health and Human development
HLPP	Bronx-Lebanon Hospital Center Health Care System<br>United Healthcare Foundation
BLISS	Lottery Health Research<br>Meat and Livestock Australia<br>Karitane Products Society<br>University of Otago<br>Heinz Watties Ltd<br>Perpetual trustees<br>NZ federation of Woman's Institutes
Preventing Childhood Obesity through early guidance	National institute of Health<br>National Institute of Diabetes and Digestive and Kidney Diseases (NIDDK)<br>Arizona State University College of Nursing and Health Innovation

PRIMROSE	Swedish Research Council for Health<br>The Swedish Council for Working Life and Welfare<br>The Swedish Research Council<br>The Research and Development Committee<br>Stockholm, Uppsala and Sormland County Council<br>Regional Research Council of the Uppsala and Orebre Health Care Region<br>The Public Health Committee of Stockholm County Council<br>The Vardal Foundation<br>AFA insurance<br>The foundation of the Swedish Diabetes Society<br>The Karolinska Health Care Sciences Postgraduate School<br>The Karolinska Institute
Early Obesity Prevention:	Dannon Institute (USA)
CHAT	NSW Ministry of Health
CHALO	Grant from the National Institute on Minority Health and Health Disparities
PROBIT	Regione Vento<br>University of Verona
Samen Happie	Behavioural Science Institute, Radbound Univeristy
Family Spirit Nurture	Eunice Kennedy National Institute of Child Health and Human Development<br>Share Our Strength<br>Indian Health service<br>John Hopkins University Discovery Award
Baby's first bites	NWO - Netherlands organisation for scientific research<br>Danone Nutricia research
SPOON Guatemala	Inter-American Developmental Bank<br>The PepsiCo Foundation<br>JPO-JSF Poverty Reduction Program

	Fundazúcar
Strong futures	University of California<br>Eunice Kennedy National Institute of child health and human development
PROGESPI	FFIS (Fundacion para la Formacion e Investigacion Sanitarias de la Region de Murcia)
SCHeLTI	International Peace Maternity and Child Health Hospital affiliated to Shanghai Jiao Tong University, School of Medicine<br>University of Sherbrooke<br>University of Sherbrooke, Health Campus<br>National Science Foundation of China(NSFC)<br>Canadian Institutes of Health Research (CIHR)
SPOON Mexico	Inter-American Development Bank<br>Hospital Infantil de Mexico Federico Gomez<br>Servicios de Salud de Nayarit<br>The PepsiCo Foundation
Greenlight plus	Vanderbilt University Medical Center<br>Patient-Centered Outcomes Research Institute<br>Duke University<br>University of North Carolina, Chapel Hill<br>Stanford University<br>NYU Langone Health<br>University of Miami

Table S7. Effectiveness of trial intervention on secondary outcomes.

Study, Author, Year	Sample Size	Outcome	Reported Outcome at End of Follow Up		Effect Size
			Control Group	Intervention Group	
		**At 2 years**			
Healthy Beginnings, Wen, 2012,	N = 667	Vegetable ≥1 serving/day	83%	89%	Difference: 7, CI (1 to 13), p = 0.03
		Fruit ≥1 serving/day	93%	90%	Difference: −2, CI (−7 to 3), p = 0.43
		Food for reward	72%	62%	Difference: −9, CI (−17 to −1), p = 0.03
		Water >3 cups/day	19%	24%	Difference 6, CI (−1 to 13), p = 0.12
		Hot chips/French fries	88%	86%	Difference −1, CI (−7 to 5), p = 0.65
		Salty snack	70%	65%	Difference −5, CI (−13 to 4), p = 0.29
		Sweet snack every day	77%	73%	Difference: −4, CI (−12 to 4), p = 0.31
		Soft drink	26%	24%	Difference: −3, CI (−10 to 5), p = 0.48
		Outdoor play ≥120 min/day	61%	62%	Difference: 1, CI (−8 to 9), p = 0.9
		TV on during meal	76%	66%	Difference: −10, CI (−18 to −2), p = 0.02
		Eat dinner in front of TV	68%	56%	Difference: −12, CI (−21 to −3), p = 0.01
		Viewing TV >60 min/day	22%	14%	Difference: −8, CI (−15 to −1), p = 0.02
		**At 3.5 years**			
Healthy Beginnings, Wen, 2015,		Vegetable ≥2	45.1%	44.5%	aOR 0.39 (95% CI, 0.62 to 1.45), p = 0.82
		serving/day	68.6%	69.7%	aOR 1.11 (95% CI, 0.71 to 1.74), p = 0.65
		Fruit ≥2 serving/day	76%	81.5%	aOR 1.56 (95% CI, 0.93 to 2.62), p = 0.09
		Food for reward	70.1%	73%	aOR 1.14 (95% CI, 0.72 to 1.81), p = 0.58
		Salty snack	79.9%	80.1%	aOR 1.05 (95% CI, 0.63 to 1.75), p = 0.85

Confectionery	28.9%	33.2%	aOR 1.26 (95% CI, 0.80 to 1.99), p = 0.32
Soft drink	68.1%	66.4%	aOR 0.96 (95% CI, 0.62 to 1.48), p = 0.86
Outdoor play ≥120 min/day	73%	64.9%	aOR 0.84 (95% CI, 0.54 to 1.31), p = 0.45
TV on during meal	66.7%	64%	aOR 0.95 (95% CI, 0.62 to 1.46), p = 0.82
Eat dinner in front of TV	6.4%	11.4%	aOR 1.68 (95% CI, 0.79 to 3.58), p = 0.18
Viewing TV >60 min/day	43.3%	42.4%	aOR 0.89 (95% CI, 0.56 to 1.4), p = 0.61
**At 5 years**			
Vegetable ≥2 serving/day	67.4%	72.8%	aOR 1.31 (95% CI, 0.81 to 2.14), p = 0.27
Fruit ≥2 serving/day	65.2%	62.3%	aOR 0.97 (95% CI, 0.61 to 1.53), p = 0.89
Food for reward	67.4%	65.4%	aOR 0.95 (95% CI, 0.60 to 1.50), p = 0.83
Salty snack	86%	79.1%	aOR 0.71 (95% CI, 0.40 to 1.28), p = 0.25
Confectionery	39.3%	37.2%	aOR 0.91 (95% CI, 0.58 to 1.42), p = 0.67
Soft drink	65.2%	67.5%	aOR 1.07 (95% CI, 0.67 to 1.71), p = 0.77
Outdoor play ≥120 min/day	71.3%	70.2%	aOR 1.12 (95% CI, 0.69 to 1.80), p = 0.65
TV on during meal	55.1%	55.5%	aOR 1.15 (95% CI, 0.74 to 1.77), p = 0.54
Eat dinner in front of TV	10%	10%	aOR 0.89 (95% CI, 0.44 to 1.83), p = 0.76

		Viewing TV >60 min/day			
INFANT, Campbell, 2013	N = 457	Child diet at 20 months<br>Fruit intake (g/d)	152.9	161.2	Mean difference 13.33 (95% CI, −2.59 to 29.25), p = 0.1
		Vegetable intake (g/d)	80.8	85.3	Mean difference 6.62 (95% CI, −2.51 to 15.76), p = 0.16
		Water intake (g/d)	338.7	362.9	Mean difference 30.28 (95% CI, −3.30 to 63.87), p = 0.08
		Noncore drink intake (g/d)	25.4	23.7	Mean difference −5.56 (95% CI, −17.48 to 6.36), p = 0.36
		Sweet snack intake (g/d)	14.7	11.0	Mean difference −3.60 (95% CI, −6.34 to −0.86), p= 0.01
		Savoury snack intake (g/d)	5.8	4.8	Mean difference −1.02 (95% CI −2.82 to 0.79), p = 0.27
		Child physical activity (min/d)	236.8	224.1	Mean difference −2.03 (95% CI, −9.75 to 5.70), p = 0.61
		Television viewing (min/d)	60.6	45.5	Mean difference −17.12 (95% CI, −26.45 to −7.79), p = <0.001
INFANT, Hesketh, 2020	N = 361<br>N = 337	Child diet at 3.6 years<br>Fruit intake (g/d)	-	-	Standardised effect size 0.23 (95% CI, 0.01 to 0.45)
		Vegetable intake at 3.6 years	-	-	Standardised effect size 0.28 (95% CI, 0.05 to 0.51)
		Water intake at 3.6 years	-	-	Standardised effect size 0.41 (95% CI, 0.14 to 0.67)
		Fruit variety at 3.6 years	-	-	Standardised effect size 0.13 (95% CI, −0.10 to 0.35)
		Vegetable variety at 3.6 years	-	-	Standardised effect size 0.24 (95% CI, 0.03 to 0.45)
		Non core drinks at 3.6 years	-	-	Standardised effect size 0.08 (95% CI, −0.18 to 0.33)

Sweet snacks intake at 3.6 years	-	-	Standardised effect size −0.24 (95% CI, −0.42 to −0.07)
Savory snack intake at 3.6 years	-	-	Standardised effect size −0.06 (95% CI, −0.23 to 0.12)
Television viewing at 3.6 years	-	-	Standardised effect size −0.08 (95% CI, −0.25 to 0.09)
Sitting time at 3.6 years	-	-	Standardised effect size −0.13 (95% CI, −0.49 to 0.23)
Child physical activity at 3.6 years	-	-	Standardised effect size 0 (95% CI, −0.26 to 0.27)
Light intensity PA at 3.6 years	-	-	Standardised effect size 0.17 (95% CI, −0.11 to 0.44)
Moderate to vigorous PA at 3.6 years	-	-	Standardised effect size −0.21 (95% CI, −0.50 to 0.08)
Child diet at 5 years Fruit intake (g/d)	-	-	Standardised effect size 0.07 (95% CI, −0.14 to 0.27)
Vegetable intake at 5 years	-	-	Standardised effect size 0.11 (95% CI, −0.10 to 0.32)
Water intake at 5 years	-	-	Standardised effect size 0.19 (95% CI, −0.03 to 0.40)
Fruit variety at 5 years	-	-	Standardised effect size 0.12 (95% CI, −0.10 to 0.33)
Vegetable variety at 5 years	-	-	Standardised effect size 0.14 (95% CI, −0.06 to 0.34)
Non core drinks at 5 years	-	-	Standardised effect size −0.17 (95% CI, −0.33 to −0.00)
Sweet snacks intake at 5 years	-	-	Standardised effect size −0.26 (95% CI, −0.47 to −0.05)
Savory snack intake at 5 years	-	-	Standardised effect size 0.00 (95% CI, −0.22 to 0.23)

		Television viewing at 5 years	-	-	Standardised effect size −0.15 (95% CI, −0.33 to 0.03)
		Sitting time at 5 years	-	-	Standardised effect size −0.08 (95% CI, −0.26 to 0.10)
		Child physical activity at 5 years	-	-	Standardised effect size 0 (95% CI, −0.26 to 0.25)
		Light intensity PA at 5 years	-	-	Standardised effect size 0.15 (95% CI, −0.09 to 0.38)
		Moderate to vigorous PA at 5 years	-	-	Standardised effect size −0.16 (95% CI, −0.42 to 0.18)
NOURISH, Daniels, 2013<br>Response to refusal of familiar foods	N = 698<br>* N = 466	Insist child eats it<br>At 2 years	37 (90)	18 (39)	$p < 0.001$
	* N = 464	Offer milk drink instead<br>At 2 years	22 (53)	14 (30)	$p = 0.022$
	* N = 465	Offer liked food instead<br>At 2 years	78 (189)	63 (140)	$p = 0.001$
	* N = 466	Encourage to eat: turn mealtime into game<br>At 2 years	57 (139)	21 (47)	$p < 0.001$
	* N = 465	Offer food reward<br>At 2 years	31 (75)	9 (19)	$p < 0.001$
	* N = 464	Encourage to eat: offer nonfood reward<br>At 2 years	27 (65)	18 (39)	$p = 0.026$
	* N = 464	Accept that child may not be hungry; take food away<br>At 2 years	91 (222)	96 (213)	$p = 0.033$
Response to refusal of unfamiliar foods (neophobia)	* N = 457	Assume child dislikes; do not offer again<br>At 2 years	13 (32)	5 (11)	$p = 0.033$

	* N = 456	Disguise food<br>At 2 years	65 (156)	45 (98)	p < 0.001
	* N = 462	Offer with liked food<br>At 2 years	94 (229)	94 (206)	p = 0.99
	* N = 465	Times offered a food before deciding whether liked (≤ 6 times)<br>At 2 years	35 (87)	72 (159)	p < 0.001
NOURISH, Daniels, 2015	* N = 390	Insist child eats it at 5 years	65 (126)	48 (94)	p = 0.001
Response to refusal of familiar foods	* N = 391	Offer milk drink instead at 5 years	6 (11)	3 (6)	p = 0.22
	* N = 389	Offer liked food instead<br>at 5 years	41 (78)	37 (72)	p = 0.47
	* N = 391	Encourage to eat<br>Feed child with spoon/fork at 5 years	53 (103)	42 (84)	p = 0.034
	* N = 390	Offer food reward<br>at 5 years	63 (120)	42 (83)	p < 0.001
	* N = 390	Offer non-food reward at 5 years	39 (74)	29 (58)	p = 0.055
Responsive feeding strategies	* N = 391	Offer no food until next usual meal/snack time at 5 years	64 (123)	77 (152)	p = 0.006
	* N = 389	Accept that child may not be hungry; take food away at 5 years	79 (152)	88 (173)	p = 0.014
Response to refusal of unfamiliar foods (neophobia)	* N = 382	Assume child dislikes; do not offer again at 5 years	14 (27)	13 (25)	p = 0.88
	* N = 382	Disguise food at 5 years	53 (102)	41 (78)	p = 0.018
	* N = 391	Offer with liked food at 5 years	92 (178)	93 (184)	p = 0.85

		Times offered a food before deciding whether liked (≤6 times) at 5 years	55 (107)	39 (77)	p = 0.002
Feeding Young Children Study, Bonuck, 2013	N = 135	Bottle frequency (any use, %)	44%	33%	11% difference in prevalence, p = 0.25
POI.nz, Fangupo, 2015	N = 495	Offered 2 fruits a day (%)	Control + sleep 94%	FAB + Sleep 96%	RR 1.02 (95% CI, 0.98 to 1.06), p = 0.455
		Offered 2 vegetables a day (%)	Control + sleep 96%	FAB + Sleep 98%	RR 1.02 (95% CI, 0.99 to 1.05), p = 0.282
		Used a cup, not bottle (%)	Control + sleep 47%	FAB + Sleep 53%	RR 1.13 (95% CI, 0.95 to 1.35), p = 0.16
		Daily breakfast (%)	Control + sleep 91%	FAB + Sleep 93%	RR 1.02 (95% CI, 0.97 to 1.08), p = 0.375
		Family dinner at the table (%)	Control + sleep 66%	FAB + Sleep 59%	RR 0.9 (95% CI, 0.79 to 1.04), p = 0.146
		Did not eat meals in front of the TV (%)	Control + sleep 15%	FAB + Sleep 17%	RR 1.16 (95% CI, 0.78 to 1.74), p = 0.464
	N = 502	Household fruit and vegetable availability (mean)	Control + sleep 31	FAB + Sleep 32	RR 0.99 (95% CI, −0.5 to 2.48), p = 0.194
		Number of obesogenic foods in household (mean number)	Control + sleep 21	FAB + Sleep 21	RR 0.39 (95% CI, −0.88 to 1.66), p = 0.39
POI.nz, Taylor, 2018	N = 592	Night sleep, duration (hour)	Controls 11.6	FAB 11.5 Sleep 11.6 Combo 11.5	p = 0.74
		Night awakenings (number per night)	Controls 1.6	FAB 1.7 Sleep 1.6 Combo 1.5	p = 0.66
	N = 602	Bedtime resistance score (mean occurrence)	Controls 0.13	FAB 0.15 Sleep 0.11 Combo 0.12	p = 0.011
	N = 351	Light to vigorous physical activity (mins per day)	Controls 231	FAB 240 Sleep 232 Combo 236	p = 0.63

BeeBOFT, Grieken, 2017	N = 1543	Health related behaviours: Breakfast daily (%)	96.7%	98.3%	1.6% difference, p = 0.03
		Activity and outside play (hours/day, mean SD)	2.56	2.68	Mean SD difference 0.12, p = 1.9
		Sweetened sugar beverage consumption (glasses/day, mean)	2.31	2.10	Mean difference −0.21 glasses per day, p = 0.003
		Television/computer time (hours/day mean)	1.22	1.05	Mean difference −0.17 h/day, p < 0.001
INSIGHT, Adams, 2018	N = 279	Met 2012 AAP screen time guidelines			
		44 weeks	30.2	53	p < 0.01
		1.5 years	15.9	23.5	p = 0.15
		2.5 years	59.8	60.9	p = 0.87
		Television on during infant meals			
		44 weeks	45.7%	32.5%	p = 0.04
		1.5 years	68.1%	48.7%	p < 0.01
		2.5 years	78.4%	66.4%	p = 0.05
		Children engagement in daily outdoor play at 2 years	15.1%	30.0%	p = 0.01
		Dietary intake of children at 1 year			
		salty snacks	20.3%	9.8%	p = 0.03
		vegetables daily	89.0%	95.9%	p = 0.049
INSIGHT, Savage, 2018	N = 279	Use of feeding to soothe			
		Context based			
		8 weeks	2.83	2.57	p = 0.008
		16 weeks	2.76	2.5	p = 0.009
		32 weeks	2.43	2	p < 0.0001
		44 weeks	2.56	2.16	p < 0.0001
		Emotion based			
		8 weeks	2.01	1.78	p = 0.07
		16 weeks	1.9	1.57	p = 0.002
		32 weeks	1.65	1.36	p = 0.0003
		44 weeks	1.62	1.31	p = 0.0001

		Adding cereal to bottle			
		8 weeks	1.8%	5.30%	CI (95% 0.6–15.6), p = 0.18
		20 weeks	20.00%	8.40%	CI (95% 0.2–0.8), p = 0.01
		32 weeks	14.00%	10.20%	CI (95% 0.3–1.6), p = 0.38
		Put child to bed with bottle/sippy cup			
		8 weeks	2.8%	0%	p = 0.95
		20 weeks	2.80%	1.70%	CI (95% 0.1–3.6), p = 0.56
		32 weeks	7.80%	5.30%	CI (95% 0.2–1.9), p = 0.42
		52 weeks	20.50%	10.40%	CI (95% 0.2–0.9), p = 0.03
		Night time feeds (7 pm–7 am)			
		8 weeks	3.3	3.1	p = 0.2
		20 weeks	2.2	1.8	p = 0.32
		32 weeks	1.7	1.3	p = 0.01
Starting Early Obesity Prevention Program, Gross, 2016	N = 533 * N = 456	Ever breastfed in the hospital	95.3%	95.9%	OR 1.16 (95% CI, 0.47 to 2.85), p = 0.82
		Exclusive BF in the hospital	31.1%	37.1%	OR 1.31 (95% CI, 0.89 to 1.93), p = 0.20
		Exclusive BF leaving the hospital	37.9%	45.7%	OR 1.38 (95% CI, 0.95 to 2.01), p = 0.11
		Any BM at 3 months	80.4%	83.3%	OR 1.21 (95% CI, 0.75 to 1.95), p = 0.47
		Exclusive BM at 3 months	23.4%	33.0%	OR 1.61 (95% CI, 1.07 to 2.44), p = 0.03
		100% BM on 24 h diet recall	33%	42.7%	OR 1.51 (95% CI, 1.03 to 2.21), p = 0.04
		Breastfeeding intensity continuous score (mean)	59.7	67.7	Mean difference −8.0 (95% CI, −15.3 to −0.75), p = 0.03
		Ever gave BM and formula at same feed per day	31.1%	22.4%	OR 0.64 (95% CI, 0.36 to 1.15), p = 0.15

		Introduced tea, water, juice or cereal in bottle at 3 months old	16.7%	6.3%	OR 0.34 (95% CI, 0.18 to 0.64), p = 0.001
		Total maternal infant feeding knowledge	9.8	10.3	Mean difference 0.51 (95% CI, 0.19 to 0.83), p = 0.002
Starting Early Obesity Prevention Program, Gross, 2017	N = 533 * N = 456	Tummy time (ever)	78.9%	86.4%	OR 1.71 (95% CI, 1.04 to 2.8), p = 0.04
		Tummy time (daily)	49.6%	50.5%	OR 1.04 (95% CI, 0.72 to 1.5), p = 0.93
		Tummy time on the floor (ever	24.1%	40.7%	OR 2.16 (95% CI, 1.44 to 3.23), p < 0.001
		Tummy time mostly on the floor	5.2%	11.8%	OR 2.44 (95% CI, 1.2 to 4.98), p = 0.02
		Mean tummy time per day	1.87	1.96	Mean difference −0.09 (95% CI, −0.46 to −0.28), p = 0.64
		Mean infant age (weeks) for starting tummy time (SD)	6.90	6.62	Mean difference 0.28 (95% CI, −0.75 to 1.31), p = 0.60
		Unrestrained floor time (ever	28.9%	40.6%	OR 1.69 (95% CI, 1.14 to 2.49), p = 0.01
		Restricted time (ever)	85.3%	85.4%	OR 1.00 (95% CI, 0.60 to 1.69), p = 1.00
		Restricted time (60 min or more)	58.6%	54.3%	OR 0.84 (95% CI, 0.58 to 1.22), p = 0.39
		Infant bouncy seat (ever)	57.5%	61.2%	OR 0.86 (95% CI, 0.59 to 1.25), p = 0.45
		Indoor baby swing (ever)	20.7%	20.4%	OR 0.98 (95% CI, 0.62 to 1.55), p = 1.00
		Car seat when not in a car (ever)	16.4%	9.5%	OR 0.54 (95% CI, 0.30 to 0.95), p = 0.04
BLISS, Taylor, 2017	N = 160	Energy self-regulation (mean SD of scale 1–5 based on parental response	4.03	4.01	Mean difference −0.04 (95% CI, −0.29 to 0.21)
		Satiety response (mean SD of scale 1–5 based on parental response)	3.23	3.01	Mean difference −0.24 (95% CI, −0.41 to −0.07)
		Food responsiveness (mean SD of scale 1–5 based on parental response)	2.41	2.51	Mean difference 0.12 (−0.09 to 0.34)

		Food fussiness (mean SD of scale 1–5 based on parental response)	2.61	2.43	Mean difference −0.18 (95% CI, −0.40 to 0.05)
	N = 113	Child enjoyment of food (mean SD of scale 1–5 based on parental response)	3.84	4.07	Mean difference 0.24 (95% CI, 0.05 to 0.43)
		Energy intake (per day), kj	4084	4026	Mean difference 143 (CI 95%, −241 to 526)
PRIMROSE, Doring, 2016	N = 1148	Children's eating habits Fruits (t/d)	1.1 (0.03)	1.1(0.03)	0.01(−0.09 to 0.11), p = 0.78
		Children's eating habits Vegetables (t/d)	0.9 (0.03)	1.0 (0.03)	0.13 (0.04 to 0.22), p = 0.01
		Children's eating habits Fish (t/wk)	1.5 (0.06)	1.6 (0.06)	0.10 (−0.06 to 0.27), p = 0.21
		Children's eating habits French fries (t/mo)	1.8 (0.07)	1.5 (0.07)	−0.37 (−0.58 to −0.17), p < 0.001
		Children's eating habits Sugared drinks (t/wk)	2.7 (0.15)	2.2 (0.18)	−0.49 (−0.97 to −0.15), p = 0.04
		Discretionary calories (t/wk)	5.9 (0.12)	5.3 (0.17)	−0.60 (−1.01 to −0.18), p= 0.01
		Mothers' eating habits —Fruits (t/d)	1.1 (0.04)	1.2 (0.03)	0.07 (−0.04 to 0.18), p = 0.22
		Mothers' eating habits —Vegetables (t/d)	1.3 (0.04)	1.3 (0.06)	0.10 (−0.02 to 0.21), p = 0.10
		Mothers' eating habits —Fish (t/wk)	1.8 (0.07)	2.0 (0.07)	0.18 (−0.01 to 0.38), p = 0.07
		Mothers' eating habits —French fries (t/mo)	1.7 (0.10)	1.4 (0.08)	−0.33 (−0.58 to −0.10), p = 0.01
		Mothers' eating habits Sugared drinks (t/wk)	1.8 (0.11)	1.5 (0.14)	−0.26 (−0.60 to 0.08), p = 0.13
		Mothers' eating habits Discretionary calories (t/wk)	5.9(0.12)	5.3(0.17)	−1.00 (−1.72 to −0.28), p = 0.01
		Children's physical activity	51 min/day	50.6 min/day	−0.36 (−3.00 to 2.28), p = 0.81

		Mothers' physical activity	2.6 (0.04)	2.6 (0.03)	0.07 (−0.02 to 0.16), p = 0.13
Early Obesity Prevention, Schroeder, 2015	N = 232	Tricep skinfold at baseline	7.85	7.94	Mean difference 0.09
		Tricep skinfold at 12 months	8.82	9.70	Mean difference 0.88 p < 0.002
		Triceps skinfold at 24 months	8.42	8.83	Mean difference 0.41
		Triceps + subscapular skinfold at baseline	14.45	14.36	Mean difference −0.09
		Triceps + subscapular skinfold at 12 months	15.36	16.46	Mean difference 1.1 p < 0.018
		Triceps + subscapular skinfold at 24 months	14.06	14.68	Mean difference 0.62
		Use infant cereal as first complimentary food	-	-	p < 0.001 (INT less likely)
		Use stage 1 vegetables as first complimentary food	-	-	p < 0.05 (INT less likely
		Offered soda	9%	1%	p < 0.006
		Sweetened tea	8.2%	1%	p < 0.01
		Punch	5.8%	0%	p < 0.02
		Cow's milk	16.2%	2.5%	p < 0.001
		Delay introduction of drink and food other than breast milk	-	-	p < 0.05 (INT more likely)
	N = 186	Perceived feeding responsibility at 24 months	4.45	4.50	Mean difference 0.05, p = 0.930
		Perceived parent overweight at 24 months	3.28	3.15	Mean difference −0.13, p = 0.409
		Perceived child overweight at 24 months	2.89	2.98	Mean difference 0.09, p = 0.194
		Concerns about child overweight at 24 months	2.06	2.29	Mean difference 0.23, p = 0.329
		Dietary restriction at 24 months	3.44	3.77	Mean difference 0.33, p = 0.010
		Pressure to eat at 24 months	2.68	2.72	Mean difference 0.04, p = 0.939
		Monitoring at 24 months	4.13	4.41	Mean difference 0.28, p = 0.046
PROBIT, Morandi, 2019	N = 550	Feeding on demand at 12 months (%)	80%	93%	13% difference, p = 0.001

N = 468	Television viewing at age 2 years (>30 mins a day, %)	67%	73%	6% difference, p = 0.08
N = 468	Sweetened Beverage consumption at age 2 years (any, %)	57%	63%	6% difference, p = 0.16

Table S8. Risk of Bias (RoB 2.0) assessments.

Study	Design	Risk of bias
Healthy Beginnings @ 2 years	RCT	High Risk
Healthy Beginnings @ 3.5, 5 years	RCT	High Risk
Feeding Young Children Study	RCT	Some Concerns
INFANT	RCT	Low Risk
INFANT at 3 and 5	RCT	Low Risk
NOURISH at 2 years	RCT	Low Risk
NOURISH at 5 years	RCT	Low Risk
PRIMROSE	RCT	High Risk
INSIGHT at 1 year	RCT	Low Risk
INSIGHT at 3 years	RCT	Low Risk
BeeBOFT	RCT	Low Risk
BLISS	RCT	Low Risk
POI.nz	RCT	Low Risk
PROBIT	RCT	Low Risk
Early Obesity Prevention	RCT	High Risk

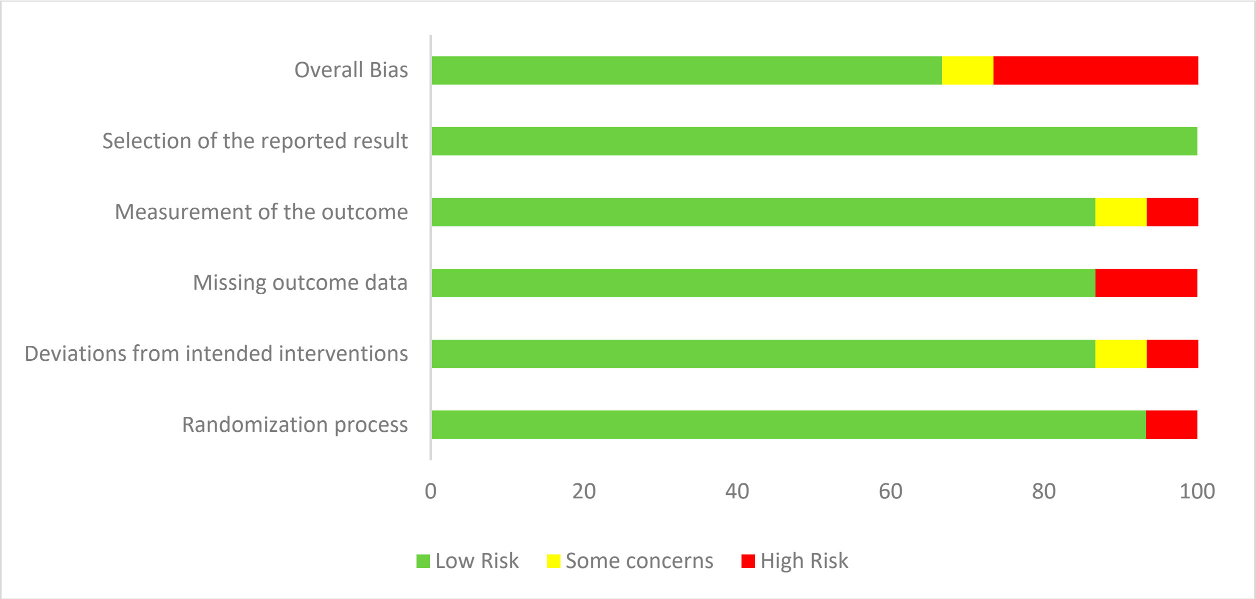

Figure S1. Risk of bias graph.
